# Estimation of Two-Dimensional Non-Symmetric Incoherently Distributed Source with L-Shape Arrays

**DOI:** 10.3390/s19051226

**Published:** 2019-03-11

**Authors:** Tao Wu, Zhenghong Deng, Yiwen Li, Zhengxin Li, Yijie Huang

**Affiliations:** 1 School of Automation, Northwestern Polytechnical University, Xi’an 710072, Shaanxi, China; dthree@nwpu.edu.cn (Z.D.); hyjrly@126.com (Y.H.); 2 Science and Technology on Combustion, Thermal-Structure and Internal Flow Laboratory, Northwestern Polytechnical University, Xi’an 710072, Shaanxi, China; lee_yiwen@nwpu.edu.cn; 3 Center for Optical Imagery Analysis and Learning, Northwestern Polytechnical University, Xi’an 710072, Shaanxi, China; zhengxinli@nwpu.edu.cn

**Keywords:** direction-of-arrival (DOA), angular spread, non-symmetric, incoherently distributed sources, L-shape arrays, expectation maximization

## Abstract

In the field of array signal processing, distributed sources can be regarded as an assembly of point sources within a spatial distribution. In this study, a two-dimensional (2D) non-symmetric incoherently distributed (ID) source model is proposed; we explore the estimation of a 2D non-symmetric ID source using L-shape arrays. The 2D non-symmetric ID source is established by modeling the angular power density function (APDF) as a Gaussian mixture model. Estimation of the non-symmetric distributed source is proposed based on the expectation maximization (EM) framework. The proposed EM iterative framework contains three steps in the process of each circle. Firstly, the nominal azimuth and nominal elevation of each Gaussian component are obtained from the phase parts of elements in sample covariance matrices. Then the angular spreads can be solved through a one-dimensional (1D) search by the original generalized Capon estimator. Finally, weights of each Gaussian component are obtained by solving the least-squares estimator. Simulations are conducted to verify the effectiveness of the estimation technique.

## 1. Introduction

In array signal processing, applications such as wireless communications, radar and underwater acoustics, point source models (assuming that signals impinging into receive arrays are from point sources) are commonly used, which can simplify calculations. In the real surroundings of radar and sonar systems, because of multipath propagation between receive arrays and targets, especially when the distances of targets and receive arrays are short, the spatial scatterers of targets cannot be ignored, assumptive condition of point source is no longer valid and point source models cannot characterize sources effectively, which should be described by distributed source models [1]. Distributed sources can be regarded as an assembly of point sources within a spatial distribution. The shape of spatial distribution is related to geometry and surface property of a target, for instance, in underwater detection. Considering multipath propagation and the surface feature of targets, distributed source models are more appropriate in near field of radar or sonar detection. 

Distributed sources may be classified into coherently distributed (CD) sources or incoherently distributed (ID) sources [1]. Characterized by deterministic angular signal distribution function (ASDF) representing spatial distribution, CD sources assume that scatterers within a source are coherent. On the contrary, scatterers of an ID source are supposed to be uncorrelated. Spatial distributions of ID sources are characterized by angular power density function (APDF). 

Spatial distribution of a distributed source, both ASDF and APDF, can be generally modeled as Gaussian, uniform or any other distribution. Parameters of ASDF and APDF contain nominal angles and angular spreads. Nominal angles can also be expressed as nominal direction-of-arrival (DOA) representing the center of targets. Nominal spreads denote the spatial extension of targets. Traditional estimation of a distributed source mainly involves estimation of DOA and angular spreads, which are implied in ASDF or APDF.

For CD sources, representative achievements of parameters estimation are deriving the rotation invariance relation with respect to different array configuration by virtue of Taylor series expansions [2,3,4,5,6,7,8,9]. Performance of distributed signal parameter estimator (DSPE) algorithm is analyzed in [10] and the performance of multiple signal classification (MUSIC) is analyzed in [11]. In this paper, the modeling and estimation of an ID source are considered.

Investigators for ID sources have been developed from classical point sources estimation techniques. DSPE [1] and dispersed signal parametric estimation (DISPARE) [12] have been developed from MUSIC, both of which involve a two-dimensional (2D) spectral search. In [13], an estimator extended from estimation of signal parameters via rotational invariance techniques (ESPRIT) has been proposed for ID sources, which use the total least square-ESPRIT (TLS-ESPRIT) algorithm to estimate the nominal DOAs of sources firstly, and then the angular spreads are estimated using the central moments of the distribution. Generalizations of Capon’s methods have been proposed in [14,15,16], which involve a two-dimensional (2D) spectral search besides a high-order matrix inversion. The maximum likelihood (ML) approach [17,18,19] has better accuracy but leads to a multidimensional nonlinear optimization requiring high computational complexity. Developed from least squares estimators, covariance matching estimation techniques (COMET) [20,21,22,23,24] have lower computational complexity than ML but with the same large sample behavior. Applying sparse representation to first-order Taylor expansion of steering vectors, in the case of small angular spreads, the authors of [25] proposed an estimator via block sparse Bayesian learning for multiple incoherently distributed sources, which has presented better accuracy under fewer snapshots.

The aforementioned methods consider sources as one-dimensional (1D) ID models, which have two parameters: the nominal DOA and angular spread. However, impinging signals and arrays sensors are not in the same plane practically. 2D distributed source models characterized by 2D DOA and 2D angular spread should be more reasonable, which usually contain four parameters: nominal azimuth, nominal elevation, azimuth angular spread and elevation angular spread. Including more parameters, there have been relatively few studies on estimation of 2D ID sources. The authors of [26] have proposed an extension of COMET for 2D ID sources, which employs alternating projection principle [27] and estimates 2D DOAs and 2D angular spreads separating source powers and noise variances to reduce complexity. Based on double parallel uniform linear arrays, the authors of [28] have proposed a TLS-ESPRIT like approach for 2D DOAs, where estimation of nominal elevations is firstly implemented via rotational invariance relations based on the diffused steering vectors derived from the first-order Taylor series expansions, then the nominal azimuth is estimated by 1D searching. Authors of [29] have proposed a (ESPRIT) like algorithm for 2D ID sources based on uniform rectangular arrays. Receive vectors of arrays in [29] are described by generalized steering vector, which is composed of nominal steering vector and its first-order partial derivatives along the direction of elevation and azimuth. Nominal angles are estimated through rotational invariance relations of generalized steering vectors, which are derived under the assumption of small angular spreads and small distance between sensors. Generally, DOA estimation of ID sources by virtue of diffused steering vectors or generalized steering vectors are derived from the first-order Taylor series expansions under the assumption of small angular spreads [26] or the assumption of both small angular spreads and small distance between sensors [28,29]. Though different methods based on different arrays have analyzed the influence of angular spreads of Gaussian or uniform ID sources on DOA estimation, what is a satisfactory accuracy respect to a real specific target has not been carried out until now. The assumption that distance between sensors is far less than wavelength of the signal is applicable to few specific detection areas, such as underwater low frequency detection.

All the distributed sources estimation techniques mentioned above assume that the shape of sources is symmetric. Nevertheless, scatterers are distributed irregularly within targets and the surface of targets is generally non-symmetric. Models of 1D non-symmetric ID sources have been proposed according to the principle that a non-symmetric distribution can be constructed by symmetric distributions. The authors of [30] have proposed a non-symmetric ID source model, where the shape of APDF can be figured via the variation of the ratio between two Gaussian distributions. The authors of [31] have employed the Gaussian mixture model to characterize the 1D non-symmetric ID sources and proposed a COMET algorithm embedded in expectation maximization (EM) framework [32,33] to estimate the 1D non-symmetric APDF. Containing more parameters, estimation for a non-symmetric ID source mainly lies in estimation of the non-symmetric APDF, which is different from estimation of a symmetric ID source.

To the best of our knowledge, there are no algorithms for 2D non-symmetric distributed sources. In this paper, we are concerned with modeling and estimation of a 2D non-symmetric ID source. As the principle that symmetric distributions can form a non-symmetric distribution is also true in the case of 2D, we present a 2D non-symmetric ID source model by constituting APDF with the 2D Gaussian mixture model. The authors of [34] have explored Cramer-Rao bound of L-shape arrays composed of two orthogonal ULAs and shown that such arrays have better accuracy potential than traditional cross arrays using same number of sensors with respect to point sources. Utilizing the propagator method, the authors of [35] have said that L-shape arrays composed of two orthogonal ULAs with less number of elements can estimate better than parallel shape arrays using the same method. Several DOA estimation methods [36,37,38,39] have been developed based on a point source model with L-shaped array placed in *xoz* or *xoy* plane. Estimation for a 2D non-symmetric ID source is proposed under EM framework based on L-shape arrays. The general EM framework is to get the best parameters by maximization of the likelihood function during the iteration process. As a 2D non-symmetric ID source has more parameters than those of an ID symmetric source; maximization of the likelihood function makes it difficult to apply for a 2D non-symmetric ID source because of nonlinear and high dimensional property. In our method, parameters are obtained successively in each EM cycle. Deducing the covariance matrices via the first-order Taylor series expansion of the steering vectors in each Gaussian component, we find that the nominal DOA parameters are related to the phase parts in elements of covariance matrices. Accordingly, nominal azimuth and nominal elevation of each Gaussian component can be obtained by the sample covariance matrices. Then angular spreads can be searched through the original Generalization of Capon’s estimator base on DOA parameters. Weights are estimated by a least squares fit of theoretical covariance and sample covariance.

The rest of this paper is organized as follows. Section 2 formulates a 2D non-symmetric ID source model and describes the received signal vectors and covariance matrix under L-shape array. Section 3 details the proposed algorithm of estimation for parameters of the 2D non-symmetric ID source. Section 4 shows numerical simulations to validate the proposed estimation method. Section 5 draws the conclusion of this paper.

## 2. Distributed Source Model

**Figure 1:**
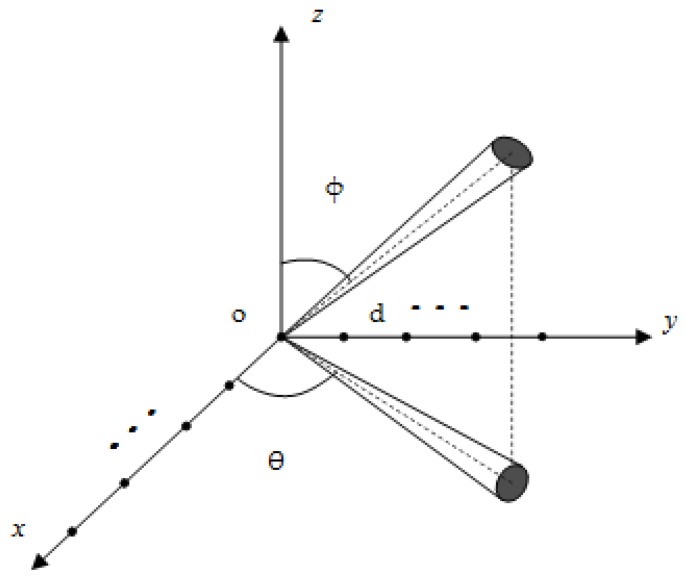
The L-shape arrays configuration.

Figure 1 shows the L-shape arrays configuration, which uses the *xoy* plane. Array X is composed of sensors on *x* axis, while sensors on *y* axis constitute array Y. Each linear array consists of *K* sensor elements separated by *d* meters, and the two linear arrays share an origin sensor. Suppose that there is a stationary narrow-band 2D ID source with azimuth angle *θ* and elevation angle *φ* distributed in a spatial distribution. *θ* ∈ [0, π/2], *φ* ∈ [0, π/2]. *λ* is the wavelength of the signal impinging into the array sensors. The *K* × 1 dimensional received signal vectors of the arrays X and Y can be expressed as follows:

(1) $$x(t)={\left[x_{1}(t),x_{2}(t),\cdots ,x_{k}(t),\cdots ,x_{K}(t)\right]}^{T},$$

(2) $$y(t)={\left[y_{1}(t),y_{2}(t),\cdots ,y_{k}(t),\cdots ,y_{K}(t)\right]}^{T},$$

where (•)*^T^* denotes the transpose. *x_k_*(t) and *y_k_*(t) are the signal received by *k*th sensors in arrays X and Y, which can be expressed as follows:

(3) $$x_{k}(t)=s(t)\sum_{l=1}^{L}\alpha_{l}(t)e^{j2\pi d(k-1)\cos\theta_{l}\sin\varphi_{l}/\lambda }+n_{xk}(t),$$

(4) $$y_{k}(t)=s(t)\sum_{l=1}^{L}\alpha_{l}(t)e^{j2\pi d(k-1)\sin\theta_{l}\sin\varphi_{l}/\lambda }+n_{yk}(t).$$

An ID source means that different scatterers from one target generate uncorrelated multipath signals. *s*(*t*) in Equations (3) and (4) is the impinging signal into the target. *L* is number of scatterers. *n_xk_*(t) and *n_yk_*(t) are noises received. *α_l_*(*t*) is random complex ray gain of the *l*th scatterer. The random complex ray gain *α_l_*(*t*) is supposed to be white and independent from snapshot to snapshot as well as from scatterer to scatterer, which has the following relationship:

(5) $$\left\{ \begin{array}{l} E[\alpha_{l}(t)\alpha_{l}(t^{\prime })]=0\\ E[\alpha_{l}(t)\alpha_{l^{\prime }}^{*}(t^{\prime })]=\frac{\sigma_{\alpha }}{L}\delta (l-l^{\prime })\delta (t-t^{\prime }) \end{array}\right.,$$

where |*α_l_*(*t*)|^2^ = *σ_α_*/*L*, *δ*(•) is the Kronecker delta function, (•)^*^ denotes the conjugate operator. Define **n***_x_*(*t*) and **n***_y_*(*t*), which are the *K* × 1 dimensional additive noise vectors of arrays X and Y; they can be written as:

(6) $$n_{x}(t)=\left[n_{x1}(t),n_{x2}(t),\cdots ,n_{xk}(t),\cdots ,n_{xK}(t)\right],$$

(7) $$n_{y}(t)=\left[n_{y1}(t),n_{y2}(t),\cdots ,n_{yk}(t),\cdots ,n_{yK}(t)\right].$$

**n***_x_*(*t*) and **n***_y_*(*t*) can be combined into

(8) $$n(t)=\left[\begin{array}{l} n_{x}(t)\\ n_{y}(t) \end{array}\right].$$

The noise is assumed to be uncorrelated with signal and uncorrelated between sensors as well as Gaussian white with zero mean:

(9) $$E[n(t)n^{H}(t\prime )]=\rho I_{2K}\delta (t-t\prime ),$$

(10) $$E[n(t)n^{T}(t\prime )]=0\;\forall t,t\prime \;,$$

where *ρ* is the power of noise, **I**_2*K*_ is the 2*K* × 2*K* identity matrix, (•)*^H^* denotes the Hermitian transpose. 

Combine **x**(*t*) and **y**(*t*) into:

(11) $$z(t)=\left[\begin{array}{l} x(t)\\ y(t) \end{array}\right].$$

The signal is assumed to be uncorrelated with the noise; the covariance matrix of **z**(*t*) can be written as follows:

(12) $$R_{z}=E[z(t)z^{H}(t)]=\left[\begin{array}{cc} R_{x} & R_{xy}\\ R_{yx} & R_{x} \end{array}\right]=\left[\begin{array}{cc} E[x(t)x^{H}(t)] & E[x(t)y^{H}(t)]\\ E[y(t)x^{H}(t)] & E[y(t)y^{H}(t)] \end{array}\right].$$

The concept of APDF reflecting the distribution of scatterers of a source can be traced back to references [1] and [19], which can be approximately modeled as 2D Gaussian and uniform or any other distribution function according to the different characteristic of ID sources. Denote *f*(*θ*,*φ*) as APDF of an ID source. The (*k*,*h*)th noise free element of covariance matrix **R***_x_* is given by (proof can be seen in Appendix A):

(13) $$\begin{array}{c} {\left[R_{x}\right]}_{kh}=E[x_{k}(t)x_{h}^{*}(t)]={\left|s(t)\right|}^{2}E\left[\sum_{l=1}^{L}\sum_{l^{\prime }=1}^{L}\alpha_{l}(t)e^{j2\pi d(k-1)\cos\theta_{l}\sin\varphi_{l}/\lambda }\alpha_{l^{\prime }}^{*}(t)e^{-j2\pi d(h-1)\cos\theta_{l^{\prime }}\sin\varphi_{l^{\prime }}/\lambda }\right]\\ =P\iint e^{j2\pi d(k-h)\cos\theta \sin\varphi /\lambda }f(\theta ,\varphi )d\theta d\varphi \end{array}.$$

where *P* = σ_α_|*s*(t)|^2^ is the received power of the target. Define **a**(*θ*,*φ*) and **b**(*θ*,*φ*) are *K* × 1 dimensional steering vectors of arrays X and Y, which can be written as follows:

(14) $$a(\theta ,\varphi )={\left[1,e^{j2\pi d\cos\theta \sin\varphi /\lambda },\cdots e^{j2\pi (K-1)d\cos\theta \sin\varphi /\lambda }\right]}^{T},$$

(15) $$b(\theta ,\varphi )={\left[1,e^{j2\pi d\sin\theta \sin\varphi /\lambda },\cdots e^{j2\pi (K-1)d\sin\theta \sin\varphi /\lambda }\right]}^{T}.$$

Thus, the covariance matrices **R***_x_*, **R***_y_*, **R***_xy_* and **R***_yx_* can be expressed as follows (proof is in Appendix B):

(16) $$\left\{ \begin{array}{l} R_{x}=P\iint f(\theta ,\varphi )a(\theta ,\varphi )a^{H}(\theta ,\varphi )d\theta d\varphi +\rho I_{K}\\ R_{y}=P\iint f(\theta ,\varphi )b(\theta ,\varphi )b^{H}(\theta ,\varphi )d\theta d\varphi +\rho I_{K}\\ R_{xy}=P\iint f(\theta ,\varphi )a(\theta ,\varphi )b^{H}(\theta ,\varphi )d\theta d\varphi \\ R_{yx}=P\iint f(\theta ,\varphi )b(\theta ,\varphi )a^{H}(\theta ,\varphi )d\theta d\varphi \end{array}\right..$$

In this study, the Gaussian mixture model is used for angular power density of the distributed source in order to express the non-symmetric distribution, so the APDF of a non-symmetric ID source can be expressed as follows:

(17) $$f(\theta ,\varphi )\approx \sum_{i=1}^{q}w_{i}g(\theta ,\varphi ;u_{i})=\sum_{i=1}^{q}w_{i}\frac{1}{2\pi \sigma_{\theta i}\sigma_{\varphi i}}\exp\left\{-0.5\left[{\left(\frac{\theta -\theta_{i}}{\sigma_{\theta i}}\right)}^{2}+{\left(\frac{\varphi -\varphi_{i}}{\sigma_{\varphi i}}\right)}^{2}\right]\right\},$$

where the APDF consists of *q* Gaussian components *g*(*θ*,*φ*; **u***_i_*) *i*= 1,2,…,*q*. **u***_i_* = [$\theta_{i}$,$\phi_{i}$,$\sigma_{\theta i}$,$\;\sigma_{\phi i}$] is the parameter set of the *i*th Gaussian component denoting the nominal azimuth, nominal elevation, azimuth angular spread, and elevation angular spread, respectively.

To normalize *f*(*θ*,*φ*), the weighting coefficient *w_i_* satisfies the following constraint:

(18) $$\sum_{i=1}^{q}w_{i}=1.$$

APDF in (17) can be shaped asymmetrically via the variation of the weighting coefficient *w_i_* and parameter set of each Gaussian component.

Combine **a**(*θ*,*φ*) and **b**(*θ*,*φ*) into:

(19) $$c(\theta ,\varphi )=\left[\begin{array}{l} a(\theta ,\varphi )\\ b(\theta ,\varphi ) \end{array}\right].$$

The summation of Gaussian mixture distributions in Equation (17) can be substituted for *f*(*θ,φ*). **R***_z_* can be expressed respectively as follows:

(20) $$R_{z}\approx \sum_{i=1}^{q}P_{i}\iint g(\theta ,\varphi ;u_{i})c(\theta ,\varphi )c^{H}(\theta ,\varphi )d\theta d\varphi +\rho I_{2K},$$

Equation (16) can be expressed as follows:

(21) $$\left\{ \begin{array}{l} R_{x}\approx \sum_{i=1}^{q}P_{i}\iint g(\theta ,\varphi ;u_{i})a(\theta ,\varphi )a^{H}(\theta ,\varphi )d\theta d\varphi +\rho I_{K}\\ R_{y}\approx \sum_{i=1}^{q}P_{i}\iint g(\theta ,\varphi ;u_{i})b(\theta ,\varphi )b^{H}(\theta ,\varphi )d\theta d\varphi +\rho I_{K}\\ R_{xy}\approx \sum_{i=1}^{q}P_{i}\iint g(\theta ,\varphi ;u_{i})a(\theta ,\varphi )b^{H}(\theta ,\varphi )d\theta d\varphi \\ R_{yx}\approx \sum_{i=1}^{q}P_{i}\iint g(\theta ,\varphi ;u_{i})b(\theta ,\varphi )a^{H}(\theta ,\varphi )d\theta d\varphi \end{array}\right.,$$

where **I***_K_* denotes the *K* × *K* identity matrix,$P_{i}=w_{i}P$ denotes power of the *i*th Gaussian component.

## 3. Proposed Method

For a 2D non-symmetric ID source, there are 5*q* unknown parameters in APDF. Compared with a 2D symmetric source, there are many more parameters to be estimated. Traditional methods such as the maximum likelihood function, COMET, and the subspace-based algorithms are difficult applications, on account of the high dimensional property of the 2D non-symmetric ID source. In this section, based on EM framework and the alternating projection principle, an iterative algorithm is proposed, which contains three steps in each iterative cycle: first, the nominal elevation angle and nominal azimuth angle of each Gaussian component are obtained utilizing feature of covariance matrices; then angular spreads are estimated by 1D searching; finally, estimation of weights are implemented though a least squares fit of theoretical and sample covariance.

### 3.1. Latent Variable Model

On the basis of the latent variable model [32,33], the observed received vectors **x**(*t*) and **y**(*t*) caused by source and noise can be considered as a combination of unobserved received vectors caused by each Gaussian component implied in APDF of (17) as well as noise accompanying each Gaussian component. **x***_i_*(*t*) and **y***_i_*(*t*) are supposed to be the *K* × 1 dimensional implied received vectors of arrays X and Y, reflecting received signal impinged by the *i*th Gaussian component and the noise accompanying the *i*th Gaussian component. According to the latent variable model, **x**(*t*) and **y**(*t*) are the observed data, which can be assumed as incomplete data; **x***_i_*(*t*) and **y***_i_*(*t*) can be assumed as complete data. The many-to-one function representing the relationship of incomplete data and complete data can be expressed as follows:

(22) $$\left\{ \begin{array}{l} x(t)=\sum_{i=1}^{q}x_{i}(t)\\ y(t)=\sum_{i=1}^{q}y_{i}(t) \end{array}\right..$$

Combine complete data **x***_i_*(*t*) and **y***_i_*(*t*) into:

(23) $$z_{i}(t)=\left[\begin{array}{c} x_{i}(t)\\ y_{i}(t) \end{array}\right].$$

Thus, we have a relationship of incomplete data **z**(*t*) and complete data **z***_i_*(*t*) as follows:

(24) $$z(t)=\sum_{i=1}^{q}z_{i}(t).$$

According to the incoherently distributed source assumption, signals from different Gaussian component are uncorrelated; so different implied received vectors **z***_i_*(*t*) are uncorrelated. Then covariance matrix of the incomplete data **z**(*t*), **R**_z_ is accordingly a summation of **R***_zi_* which is covariance matrix of the complete data **z***_i_*(*t*).*_,_* **R**_z_ can be expressed as:

(25) $$R_{z}=\sum_{i=1}^{q}R_{zi}=\sum_{i=1}^{q}E[z_{i}(t)z_{i}^{H}(t)]=\left[\begin{array}{cc} \sum_{i=1}^{q}R_{xi} & \sum_{i=1}^{q}R_{xyi}\\ \sum_{i=1}^{q}R_{yxi} & \sum_{i=1}^{q}R_{yi} \end{array}\right],$$

As **z***_i_*(*t*) is caused by the *i*th Gaussian component and its accompanied noise, covariance matrix of the complete data **R***_zi,_* can be a written as:

(26) $$R_{zi}=E[z_{i}(t)z_{i}^{H}(t)]=P_{i}\iint g(\theta ,\varphi ;u_{i})c(\theta ,\varphi )c^{H}(\theta ,\varphi )d\theta d\varphi +\rho_{i}I_{2K},$$

where *ρ_i_* is noise power of complete data **z***_i_*(*t*). From Equations (22) and (23), we find that **R***_zi_* is composed of four parts—covariance matrix of the complete data **x***_i_*(*t*) **R**_x*i*_*,* covariance matrix of the complete data **y***_i_*(*t*) **R**_y*i*_*,* covariance matrix of the complete data **x***_i_*(*t*) and **y***_i_*(*t*) **R**_xy*i*_*,* covariance matrix of the complete data **y***_i_*(*t*) and **x***_i_*(*t*) **R**_yx*i*_, which means that:

(27) $$R_{zi}=\left[\begin{array}{cc} R_{xi} & R_{xyi}\\ R_{yxi} & R_{xi} \end{array}\right]=\left[\begin{array}{cc} E[x_{i}(t)x_{i}{}^{H}(t)] & E[x_{i}(t)y_{i}{}^{H}(t)]\\ E[y_{i}(t)x_{i}{}^{H}(t)] & E[y_{i}(t)y_{i}{}^{H}(t)] \end{array}\right].$$

Denote the normalized noise-free covariance matrix of complete data **z***_i_*(*t*) as follows:

(28) $$r_{z}(u_{i})=\iint g(\theta ,\varphi ;u_{i})c(\theta ,\varphi )c^{H}(\theta ,\varphi )d\theta d\varphi =\left[\begin{array}{cc} r_{x}(u_{i}) & r_{xy}(u_{i})\\ r_{yx}(u_{i}) & r_{y}(u_{i}) \end{array}\right].$$

The noise is assumed to be distributed in complete data equally so covariance matrix of **z***_i_*(*t*), **R**_zi_ expressed by Equation (26) can also be expressed as follows:

(29) $$R_{zi}=P_{i}r_{z}(u_{i})+\frac{\rho }{q}I_{2K}.$$

**R**_x*i*_, **R**_y*i*_, **R**_yx*i*_ and **R**_yx*i*_ can be expressed as follows:

(30) $$\left[\begin{array}{cc} R_{xi} & R_{xyi}\\ R_{yxi} & R_{yi} \end{array}\right]=P_{i}\left[\begin{array}{cc} r_{x}(u_{i}) & r_{xy}(u_{i})\\ r_{yx}(u_{i}) & r_{y}(u_{i}) \end{array}\right]+\frac{\rho }{q}I_{2K}.$$

Assuming that the angular spread of both azimuth and elevation in each Gaussian component is small, *d*/*λ* is set at 1/2; sin*θ*sin*φ* and cos*θ*sin*φ* can be approximated by the first term in the Taylor series expansions. Thus, elements in the normalized noise-free signal covariance matrix of Equation (30) can be expressed as follows (proof can be seen in Appendix C):

(31) $$\left\{ \begin{array}{l} {\left[r_{x}(u_{i})\right]}_{kh}\approx e^{j\pi (k-h)\cos\theta_{i}\sin\varphi_{i}}e^{-0.5\left\{{\left[\pi \sigma_{\theta i}(k-h)\sin\theta_{i}\sin\varphi_{i}\right]}^{2}+{\left[\pi \sigma_{\varphi i}(k-h)\cos\theta_{i}\cos\varphi_{i}\right]}^{2}\right\}}\\ {\left[r_{xy}(u_{i})\right]}_{kh}\approx e^{j\pi \left[(k-1)\cos\theta_{i}\sin\varphi_{i}-(h-1)\sin\theta_{i}\sin\varphi_{i}\right]}\cdot {\left[C(u_{i})\right]}_{kh}\\ {}[r_{yx}(u_{i}){]}_{kh}\approx e^{j\pi \left[(k-1)\sin\theta_{i}\sin\varphi_{i}-(h-1)\cos\theta_{i}\sin\varphi_{i}\right]}\cdot {\left[D(u_{i})\right]}_{kh}\\ {}[r_{y}(u_{i}){]}_{kh}\approx e^{j\pi (k-h)\sin\theta_{i}\sin\varphi_{i}}e^{-0.5\left\{{\left[\pi \sigma_{\theta i}(k-h)\cos\theta_{i}\sin\varphi_{i}\right]}^{2}+{\left[\pi \sigma_{\varphi i}(k-h)\sin\theta_{i}\cos\varphi_{i}\right]}^{2}\right\}}\\ {\left[C(u_{i})\right]}_{kh}=e^{-0.5\left\{\pi^{2}\sigma^{2}{}_{\theta i}{\left[(k-1)\sin\theta_{i}\sin\varphi_{i}+(h-1)\cos\theta_{i}\sin\varphi_{i}\right]}^{2}+\pi^{2}\sigma^{2}{}_{\varphi i}{\left[(k-1)\cos\theta_{i}\cos\varphi_{i}-(h-1)\sin\theta_{i}\cos\varphi_{i}\right]}^{2}\right\}}\\ {}[D(u_{i}){]}_{kh}=e^{-0.5\left\{\pi^{2}\sigma^{2}{}_{\theta i}{\left[(k-1)\cos\theta_{i}\sin\varphi_{i}+(h-1)\sin\theta_{i}\sin\varphi_{i}\right]}^{2}+\pi^{2}\sigma^{2}{}_{\varphi i}{\left[(k-1)\sin\theta_{i}\cos\varphi_{i}-(h-1)\cos\theta_{i}\cos\varphi_{i}\right]}^{2}\right\}} \end{array},\;\right.$$

where [•]*_kh_* denotes the element of the *k*th row and the *h*th column in a matrix.

### 3.2. EM Algorithm

The EM algorithm is an iteration process containing two steps in turn: an expectation step (E-step) and a maximization step (M-step). The E-step serves to obtain parameters that are implied in each Gaussian component under the condition of incomplete data and the parameters values of E-step in the last EM circle. The M-step serves to update parameters based on the data from parameters obtained in the E-step, which is usually performed by maximizing the logarithm of the likelihood function.

The sample covariance matrix of incomplete data **z**(*t*), **R**_z_ can be replaced by ${\hat{R}}_{z}$ with *N* snapshots, which is defined as follows:

(32) $${\hat{R}}_{z}=\frac{1}{N}\sum_{1}^{N}z(t)z^{H}(t).$$

${\hat{R}}_{zi}$,${\hat{R}}_{xi}$ and ${\hat{R}}_{yi}$ are the estimated sample covariance matrix of complete data **z***_i_*(*t*), **x***_i_*(*t*) and **y***_i_*(*t*). As ${\hat{R}}_{zi}$ is a sufficient statistic of unknown parameters *w_i_*, *𝜃_i_*, *𝜙_i_*, *𝜎_𝜃i_*, *𝜎_𝜙i_* and *P*, the (*m+*1)th E-step of the EM algorithm serves to calculate the expected value of sufficient statistics as follows (the proof can be seen in Appendix D):

(33) $${\hat{R}}_{zi}^{m+1}=E[{\hat{R}}_{zi}|{\hat{R}}_{z}]=R_{zi}^{m}{\left(R_{z}^{m}\right)}^{-1}{\hat{R}}_{z}{\left(R_{z}^{m}\right)}^{-1}R_{zi}^{m}+R_{zi}^{m}-R_{zi}^{m}{\left(R_{z}^{m}\right)}^{-1}R_{zi}^{m},$$

where the superscript *m* indicates the value at the *m*th iteration.

To simplify the calculation, we assume the same angular spread for both azimuth and elevation $\sigma_{i}=\sigma_{\theta i}$=$\;\sigma_{\phi i}$. The M-step will minimize the negative logarithm of the likelihood function to find the optimal parameters in the (*m* + 1)th iteration, which can be expressed as follows:

(34) $$w_{i}^{m+1},\theta_{i}^{m+1},\varphi_{i}^{m+1},\sigma_{i}^{m+1},P_{i}^{m+1}=\underset{w_{i},u_{i}}{\mathrm{argmin}}\left[L(u_{i})=\log\left|R_{zi}\right|+\mathrm{Tr}(R_{zi}^{-1}{\hat{R}}_{zi}^{m+1})\right](i=1,\cdots ,q),$$

where parameters to be estimated are implied in **R***_zi_* which can be expressed by Equations (29)–(31). Equation (34) means exploring the best (*m* + 1)th parameters of the *i*th Gaussian component based on the sample covariance matrix of the complete data ${\hat{R}}_{zi}^{m+1}$. Minimizing the Equation (34) is computationally complicated because of nonlinearity and inversion of the high-dimensional matrix; therefore, solving all the unknown parameters simultaneously is impossible. According to the principle of alternating projection, parameters can be estimated successively based on other parameters that have already been solved in M-step.

According to Equation (27), the sample covariance matrices of the complete data **x***_i_*(*t*) and **y***_i_*(*t*) can be obtained as follows:

(35) $${\hat{R}}_{xi}^{m+1}=\left[\begin{array}{cc} I_{K} & 0_{K}\\ 0_{K} & 0_{K} \end{array}\right]{\hat{R}}_{zi}^{m+1},$$

(36) $${\hat{R}}_{yi}^{m+1}=\left[\begin{array}{cc} 0_{K} & 0_{K}\\ 0_{K} & I_{K} \end{array}\right]{\hat{R}}_{zi}^{m+1},$$

where **I***_K_* is the *K* × *K* identity matrix and **0***_K_* is *K* × *K* zero matrix. 

From Equation (31), we show that the phase parts of the elements of **r***_x_*(**u***_i_*) and **r***_y_*(**u***_i_*) contain information of cos*θ_i_*sin*φ_i_* and sin*θ_i_*sin*φ_i_*, which means that we can obtain cos*θ_i_*sin*φ_i_* and sin*θ_i_*sin*φ_i_* from phase parts of **R***_xi_* and **R***_yi_*. Average phase parts of all elements in matrix ${\hat{R}}_{xi}$ and ${\hat{R}}_{yi}$ except the real diagonal elements, denote *η_i_* as cos*θ_i_*sin*φ_i_* and *υ_i_* as sin*θ_i_*sin*φ_i_*. Thus, we obtain:

(37) $$\eta_{i}^{m+1}=\frac{2}{K^{2}-K}\sum_{h=1}^{k-1}\sum_{k=2}^{K}\frac{\mathrm{angle}({\left[{\hat{R}}_{xi}^{m+1}\right]}_{kh})}{\pi (k-h)},$$

(38) $$\upsilon_{i}^{m+1}=\frac{2}{K^{2}-K}\sum_{h=1}^{k-1}\sum_{k=2}^{K}\frac{\mathrm{angle}({\left[{\hat{R}}_{yi}^{m+1}\right]}_{kh})}{\pi (k-h)},$$

where angle(•) denotes the phase of a complex number. Thus, we obtain *θ_i_* and *φ_i_* of the *i*th Gaussian component as:

(39) $$\theta_{i}^{m+1}=\arctan(\upsilon_{i}^{m+1}/\eta_{i}^{m+1}),$$

(40) $$\varphi_{i}^{m+1}=\arcsin(\sqrt{{\left(\eta_{i}^{m+1}\right)}^{2}+{\left(\upsilon_{i}^{m+1}\right)}^{2}}).$$

After the nominal azimuth $\;\theta_{i}$ and nominal azimuth angle $\phi_{i}$ are solved, the angular spread of the *i*th Gaussian components can be estimated by using the original generalized Capon estimator:

(41) $$\sigma_{i}^{m+1}=\underset{\sigma_{i}}{\mathrm{argmin}}\mu_{\max}[{\left({\hat{R}}_{zi}^{m+1}\right)}^{-1}r_{z}(\theta_{i}^{m+1},\varphi_{i}^{m+1},\sigma_{i})],$$

where *μ*_max_(•) represents the maximal eigenvalue of a matrix.

The least-squares fit of the theoretical and sample covariance can be expressed as:

(42) $$U={\left\|{\hat{R}}_{zi}-P_{i}r_{z}(u_{i})-\frac{\rho }{q}I_{2K}\right\|}_{F}^{2}.$$

Differentiating Equation (42) with respect to *P_i_* and *ρ* setting the results to zero yields the following equation:

(43) $$P_{i}^{m+1}=\frac{tr[{\hat{R}}_{zi}^{m+1}r_{z}(u_{i}^{m+1})]-tr({\hat{R}}_{zi}^{m+1})}{tr[r_{z}^{2}(u_{i}^{m+1})]-2K}.$$

At last, the weight of the *i*th Gaussian component can be obtained as follows:

(44) $$w_{i}^{m+1}=\frac{P_{i}^{m+1}}{\sum_{i}^{q}P_{i}^{m+1}}.$$

After all parameters of the Gaussian component obtained in the (*m* + 1)th M-step, $R_{zi}^{m+1}$ can be expressed by Equations (29)–(31). $R_{z}^{m+1}$ is the summation of $R_{zi}^{m+1}$ according to Equation (25). Then the (*m* + 2)th iteration can be started.

### 3.3. Complexity Analysis and Comparison

Now, we analyze the computational complexity of the proposed method in comparison with COMET [26] and DISPARE [12], which are estimators for symmetric ID sources. It is noteworthy that the original algorithm in DISPARE [12] is for 1D ID sources and can be extended for 2D ID sources. COMET [26] is a method of estimation under alternating the projection algorithm framework; its computational cost mostly consists of calculation of the sample covariance matrix and the alternating projection technique with respect to cost functions. The DISPARE method estimates DOA and angular spreads through a three-dimensional (3D) spectrum-peak searching and its computation cost is mostly made of three parts: the calculation of the sample covariance matrix, the eigendecomposition of the matrix, and a 3D searching. The computation cost of the proposed method in one EM circle mainly consists of three operations: calculation of the 2*K* × 2*K* sample covariance matrix, 1D searching for angular spread, and calculation of weights. Assume that *M* is the EM iteration number. Table 1 shows the main computational costs of three methods. From Table 1, we can clearly see if computational cost of estimation for non-symmetric distributed source is significantly higher than that of symmetric distributed source.

The stopping criterion of the EM algorithm is when all the parameters are no longer changing [30,31]. We define the iterative variation of all parameters as:

(45) $$\Delta =\frac{1}{4q}\left|\frac{\varepsilon_{\gamma }^{m}-\varepsilon_{\gamma }^{m}}{\varepsilon_{\gamma }^{m}}\right|\gamma =1,2,\cdots ,4q,$$

where $\varepsilon_{\gamma }^{m}$ represents a parameter value in the *m*th iteration. When Δ reaches a sufficiently small value, all parameters can be considered keeping stable.

Now, our algorithm can be summarized as follows

Step 1: Determine the number *q* of Gaussian components. Initialize *P, w_i_, θ_i_, φ_i_, σ_i_* ( *i* = 1,2,…,*q*).

Step 2: Compute the sample covariance matrix of incomplete data ${\hat{R}}_{z}$ using Equation (32).

Step 3: Repeat the following sub-steps for *M* times, which is a sufficiently large number or until the iterative variation of all parameters reaches the condition Δ≤0.001.

1. Compute the sample covariance matrices of complete data ${\hat{R}}_{zi}^{m}$, ${\hat{R}}_{xi}^{m}$ and ${\hat{R}}_{yi}^{m}$ using Equations (33), (35) and (36).
2. Calculate the nominal azimuth $\theta_{i}^{m+1}$ and nominal elevation $\varphi_{i}^{m+1}$ from Equations (37)–(40).
3. Calculate $r_{z}(u_{i}^{m+1})$ using Equation (27) and search angular spread $\sigma_{i}^{m+1}$ rough 1D search by Equation (41).
4. Estimate the power of each component $P_{i}^{m+1}$ using Equation (39) and calculate weight of each component $w_{i}^{m+1}$ by Equation (44).
5. Repeat sub-steps 1 to 4 for *i* = 1, 2, …, *q*.
6. Superscript *m* = *m* + 1.

It is noteworthy that the distribution of a 2D non-symmetric ID source is unknown, so the true number of Gaussian components is unknown. Estimation is performed as *q* is an initial parameter, which needs to be set artificially. Step 1 can be considered as 0th iteration of the EM cycle. *P*^0^ is supposed to equal to $\rho^{o}$, which can be set at a unit power. $\sigma_{i}^{0}$ is set at a small value initially. $w_{i}^{0}$ can be set at 1/*q*. Then, ${\hat{R}}_{zi}^{0}$ can be obtained according to Equations (29)–(31). ${\hat{R}}_{z}^{0}$ can be obtained from Equation (25). As the incomplete data **z**(*t*) is observed, ${\hat{R}}_{zi}^{1}$ can consequently be obtained from Equation (33).

## 4. Results and Discussion

In this section, we investigate the effectiveness of the proposed method though four simulation experiments. Assume that L-shape arrays have a configuration as in Figure 1 with sensors numbers *K* = 4 in both X and Y axis, *d*/*λ* is set at 1/2. *SNR* is defined as follows:

(46) $$SNR=10\log\frac{P}{\rho }.$$

Root mean squared error (*RMSE*) is used to evaluate estimation performance. The *RMSE* of the nominal angle is defined as:

(47) $$RMSE_{a}=\sqrt{\frac{1}{Mc}\sum_{\varsigma }^{Mc}{\left({\hat{\theta }}^{\varsigma }-\theta \right)}^{2}+\frac{1}{Mc}\sum_{\varsigma }^{Mc}{\left({\hat{\varphi }}^{\varsigma }-\varphi \right)}^{2}},$$

where ${\hat{\theta }}^{\varsigma }$ and ${\hat{\varphi }}^{\varsigma }$ are the estimated nominal azimuth and estimated nominal elevation of the ID source, respectively. The superscript ς denotes the estimated parameters in ςth Monte Carlo run. *Mc* is the number of Monte Carlo simulations, which is 500. *θ* and *φ* are the true nominal azimuth and nominal elevation, respectively.

We define the value corresponding to the maximum point of the APDF as the nominal angle of the non-symmetric ID source. Nominal angle can be obtained through partial derivative of the estimated APDF, according to the property of continuous distribution. 

In addition to investigation of nominal angles, estimation of the spatial distribution should be emphasized with respect to a non-symmetric distributed source. To evaluate the estimation of spatial distribution, the *RMSE* of distributed function is defined as follows:

(48) $$RMSE_{f}=\frac{1}{Mc}\sum_{\varsigma }^{Mc}\iint \sqrt{{\left[f(\theta ,\varphi ;{\hat{u}}^{\varsigma })-f(\theta ,\varphi ;u)\right]}^{2}}d\theta d\varphi ,$$

where $f(\theta ,\varphi ;{\hat{u}}^{\varsigma })$ is the estimated APDF in ςth Monte Carlo run. In this section, we compare the performances of the proposed algorithm with two traditional estimators for ID sources, the COMET [26], which can be applied for any 2D arrays, and DISPARE [12], which can be extended for a 2D case. A 2D non-symmetric ID source with APDF is constructed as follows:

(49) f(θ,φ;u)=0.2g(40,40,2.5)+0.2g(45,40,2.5)+0.2g(50,40,2.5)+0.3g(40,45,2.5)+0.1g(40,50,3),

where *g*(*a*_1_,*a*_2_,*a*_3_) denotes a Gaussian component

(50) $$g(a_{1},a_{2},a_{3})=\frac{1}{2\pi a_{3}^{2}}\exp\left\{-0.5\left[{\left(\frac{\theta -a_{1}}{a_{3}}\right)}^{2}+{\left(\frac{\varphi -a_{2}}{a_{3}}\right)}^{2}\right]\right\}.$$

**Figure 2:**
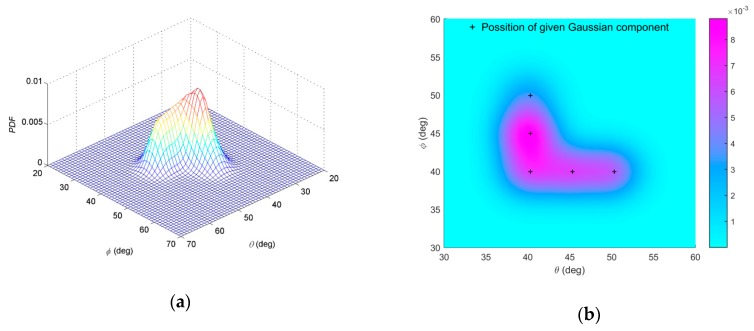
(**a**) Probability density function of APDF of the constructed non-symmetric ID source; (**b**) projection of the APDF on the *θ*−*φ* plane.

The nominal angle of the APDF can be calculated as (40°, 44.4°). Figure 2a,b shows the constructed non-symmetric APDF. The purple region of Figure 2b is projection of the constructed non-symmetric APDF on the *θ*-*φ* plane and the color bar represents measurement of probability density function (PDF). Mark + represents the central position of each Gaussian component in Equation (49). 

When estimating a 2D non-symmetric ID source, we do not know details of the non-symmetric APDF. The proposed algorithm is performed by setting initial parameters of the Gaussian mixture and iterates until the parameters no longer change. The following experiments examine the parameter variety process of each Gaussian component, different experimental conditions, number of Gaussian components, and initial positions of Gaussian components. Effectiveness of the estimated APDF can be measured though *RMSE_a_* and *RMSE_f_* .

**Figure 3:**
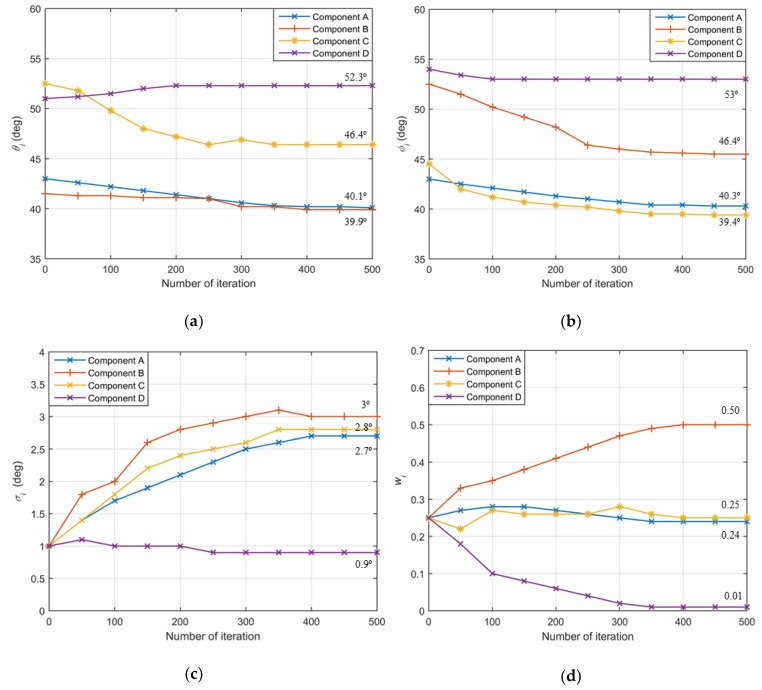
(**a**) The variety process of nominal azimuths in EM iterations; (**b**) the variety process of nominal elevations in EM iterations; (**c**) the variety process of angular spreads in EM iterations; (**d**) the variety process of weights in EM iterations.

**Figure 4:**
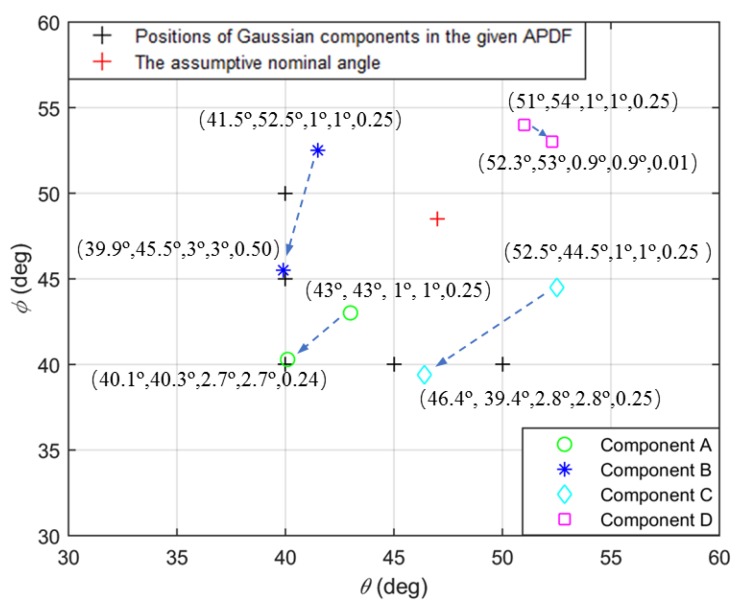
The initial and final values of each Gaussian component.

In the first example, the variety processes of Gaussian components in EM iterations are investigated. We choose four Gaussian components to estimate the source and set number of snapshots *N* = 200 and *SNR* = 15dB. As the shape of APDF of a 2D non-symmetric ID source to be estimated is unknown, we can firstly get DOA estimated by the traditional method, which is defined as an assumptive nominal angle of the 2D non-symmetric ID source. To be more representative, the initial positions of Gaussian components are set uniformly around the assumptive value with same distance to the assumptive nominal angle. Thus, the nominal azimuth and nominal elevation of four Gaussian components—A, B, C and D—are set uniformly around the value (47°,48.5°), estimated by DISPARE and set at (43°, 43°), (41.5°, 52.5°), (52.5°, 44.5°) and (51°, 54°) respectively, where the distances from initial Gaussian components to the assumptive nominal angle are all 6.8°. The initial angular spreads are set at 1°. Figure 3 shows the variety processes of parameters of each component. Figure 4 shows the initial and final values of each Gaussian component. The ordered array in parentheses ($\theta_{i}$,$\phi_{i}$,$\sigma_{\theta i}$,$\;\sigma_{\phi i}$, $w_{i}$) of Figure 4 is the parameters of the *i*th Gaussian component. The beginning of the arrow represents the initial value, while the end of the arrow is the final value. The final estimated APDF is:

(51) $$f(\theta ,\varphi ;\hat{u})=0.24g(40.1,40.3,2.7)+0.5g(39.9,46.4,3)+0.25g(46.4,40,2.8)+0.01g(39.4,45,0.9)$$

**Figure 5:**
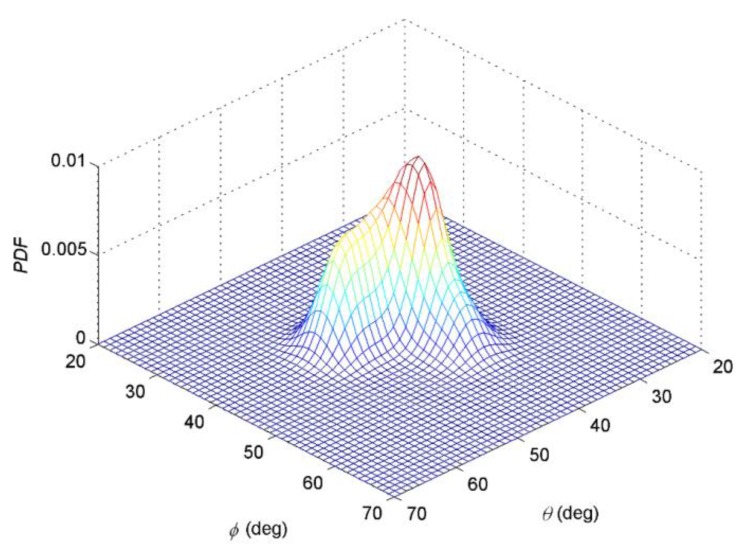
The estimation of non-symmetric APDF by the proposed method.

The nominal angle of the APDF is (39.5°, 45.9°), which is near the nominal angle of the given sources. *RMSE_a_* is 1.58° and *RMSE_f_* is 0.29. The APDF is displayed in Figure 5, which reflects the spatial non-symmetric feature of the source and is similar to the given source. It is seen that parameters will converge to certain values as a result of sufficient EM iterations. A noticeable phenomenon wherein a small weight, 0.01, developed from component D whose central position is originally far from the given source, indicates that the initial Gaussian component outside the scope of the distributed source makes hardly any contribution to the final results.

**Figure 6:**
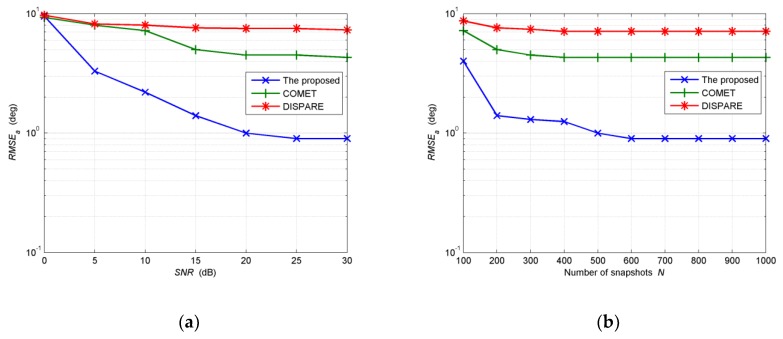
(**a**) *RMSE_a_* estimated by the three methods versus *SNR;* (**b**) *RMSE_a_* estimated by the three methods versus number of snapshots.

**Figure 7:**
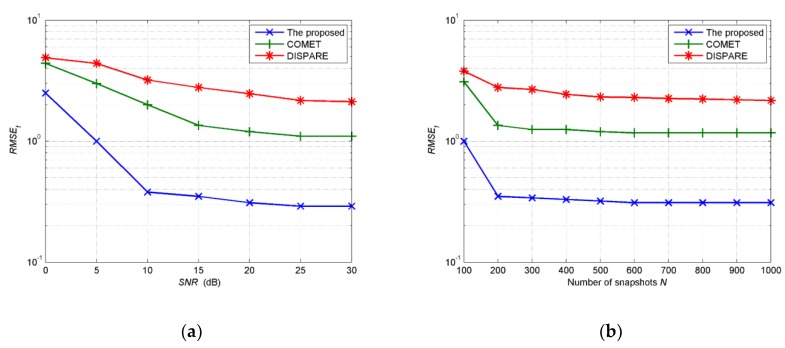
(**a**) *RMSE_f_* estimated by the three methods versus *SNR;* (**b**) *RMSE_f_* estimated by the three methods versus number of snapshots.

In the second example, we investigate the influence of *SNR* and the number of snapshots *N* on the performance of the proposed algorithm in comparison with COMET and DISPARE. The *RMSE_a_* of the proposed algorithm and COMET, as well as DISPARE at different *SNR* and different number of snapshots *N* are shown, respectively, in Figure 6a,b, while *RMSE_f_* of three methods at different *SNR* and *N* are shown, respectively, in Figure 7a,b. The EM iteration number *M* is set at 500. We also choose four Gaussian components to estimate the unknown source. The distances from initial Gaussian components to the assumptive nominal angle of the source are all set at 6.8°. The initial angular spreads are set at 1°. Then we randomly choose four positions with equal interval around the assumptive nominal angle (47°, 48.5°) in each Monte Carlo run. Five hundred independent Monte Carlo simulations are run to obtain the results. The number of snapshots *N* is set at 200 in experiments shown in Figure 6a and Figure 7a, while the *SNR* is set at 15dB in experiments shown in Figure 6b and Figure 7b. As the number of snapshots *N* or *SNR* increases, all algorithms provide better performance. Apparently, the proposed algorithm provides higher estimation accuracy than COMET and DISPARE algorithm with regard to *RMSE_a_* and *RMSE_f_*. As can be shown in Figure 6a,b, *RMSE_a_* of COMET and DISPARE reach 4.3°, 4.3°, 7.1°, 7.3°. In Figure 7a,b, we have found that the *RMSE_f_* of COMET and DISPARE reach 1.08, 1.1, 2.1, 2.3. As to *RMSE_f_*, supposing in ςth Monte Carlo trail, if the estimated APDF $f(\theta ,\varphi ;{\hat{u}}^{\varsigma })$ = 0, $\iint \sqrt{{\left[f(\theta ,\varphi ;{\hat{u}}^{\varsigma })-f(\theta ,\varphi ;u)\right]}^{2}}d\theta d\varphi$ = 1. *RMSE_f_*s estimated by COMET and DISPARE, are big errors considering distribution of function. Therefore, Figure 7a,b show that COMET and DISPARE is invalid as to the spatial distribution estimation of the given non-symmetric distributed source on account of the large errors of *RMSE_f_*.

**Figure 8:**
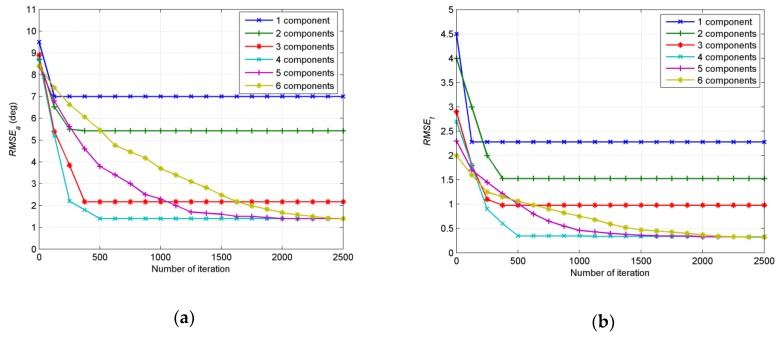
(**a**) *RMSE_a_* estimated by different number of Gaussian components; (**b**) *RMSE_f_* estimated by different number of Gaussian components.

In the third example, the influence of number of Gaussian components on performance is examined. The number of snapshots *N* = 200 and *SNR* = 15 dB. The initial angular spreads are set at 1°. When the performances of two or more Gaussian components are investigated, central positions of Gaussian components with equal interval around the assumptive nominal angle (47°, 48.5°) are randomly chosen in each Monte Carlo run; the distances from initial central positions to the assumptive nominal angle of the source are all set at 6.8°; 500 independent Monte Carlo simulations are run to obtain the result. As can be seen in Figure 8, the utilization of one Gaussian component provides a large error, which is an estimation considering sources as symmetric in essence. As the number of Gaussian components increase, *RMSE_f_* and *RMSE_a_* decrease. However, the final results of both *RMSE_f_* and *RMSE_a_* have little difference as the number changes from 3 to 6. Meanwhile, the convergence is markedly slower. It can be concluded that an increasing number of Gaussian components will provide a higher estimation accuracy, but the performance will not be notably improved as the number increase to a certain extent.

On the premise of not considering the cost of calculation, we can theoretically use any number of Gaussian components to fit a 2D non-symmetric ID source. In the third example, we examine the influence of different number of Gaussian components on estimation of a given source where the true number of Gaussian components is 5. It is found that the accuracy of estimation will not be significantly improved when *q* exceeds a certain extent. This phenomenon may be related to the shape of the given source. If the degree of asymmetry of the given 2D ID source is low, though it is composed of many Gaussian components, fewer Gaussian components can also fit the given 2D ID source well.

As the initial parameters of Gaussian components are set around assumptive value estimated by DISPARE or other methods for 2D symmetric sources, there will inevitably be Gaussian components with central positons outside the scope of the distributed source. To improve the robustness and accuracy of the algorithm, the number of Gaussian components should be set at a high level, and computing cost is also a matter of balance.

**Figure 9:**
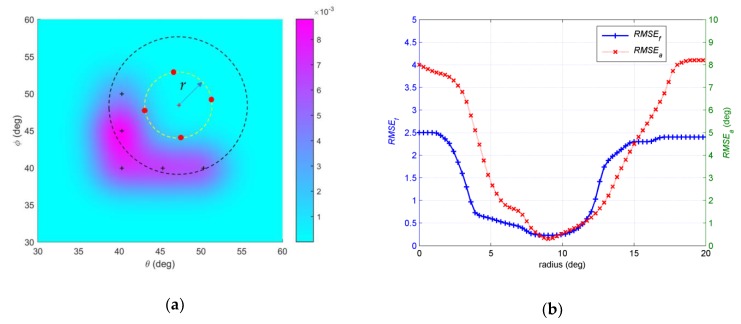
(**a**) Description of initial positions of Gaussian components and circles *r* equals 8.5°; (**b**) *RMSE_a_* and *RMSE_f_* estimated by different *r* with assumptive nominal angle (47°, 48.5°).

**Figure 10:**
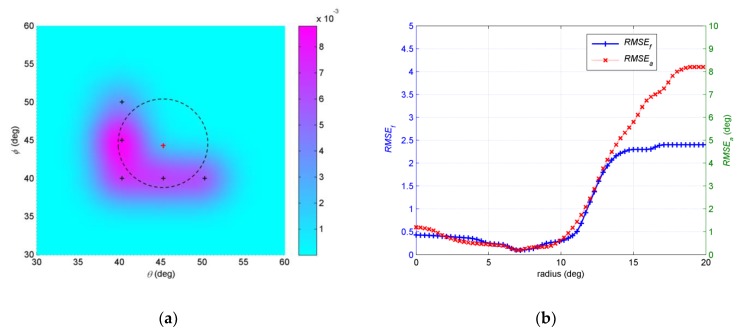
(**a**) Description of assumptive nominal angle (45°, 44°) and circles *r* equals 6.8°; (**b**) *RMSE_a_* and *RMSE_f_* estimated by different *r* with assumptive nominal angle (45°, 44°).

**Figure 11:**
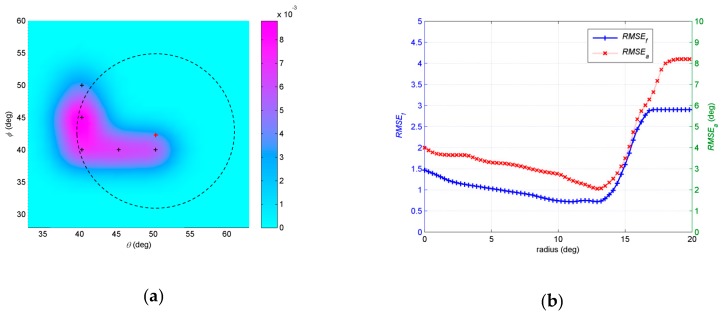
(**a**) Description of assumptive nominal angle (50°, 42°) and circles *r* equals 13.4°; (**b**) *RMSE_a_* and *RMSE_f_* estimated by different *r* with assumptive nominal angle (50°, 42°).

In the fourth example, we investigate the influence of initial positions to the final results. The number of snapshots *N* = 200 and *SNR* = 15dB. The initial angular spreads are set at 1°. The EM iteration is stopped under the condition Δ≤0.001. Three different assumptive nominal angles are considered. The first assumptive nominal angle is (47°,48.5°). Figure 9a shows that a circle is defined around the assumptive nominal angle (47°,48.5°). *r* is radius of the circle. We randomly select initial central positions of the Gaussian components, which are four points with equal interval on circle, such as the red dots in Figure 9a. 500 independent Monte Carlo simulations are run in a same circle, and then *RMSE_a_* and *RMSE_f_* are obtained with regard to each circle. We examine the radius of circle *r* changing from 0° to 20°. As shown in Figure 9b, both *RMSE_a_* and *RMSE_f_* change when the radius of the circle changes. The trends of the two curves are roughly the same. In general, as the radius increases, the estimation error decreases and then increases. When *r* is 8.5°, both *RMSE_a_* and *RMSE_f_* touch the bottom. The circle whose *r* equals 8.5° is drawn in dotted black line in Figure 9a. The second assumptive nominal angle is (45°, 44°), which is shown in Figure 10a. Setting process of initial positions is same as the first one. We examine the influence of radius of circle *r* changing on estimation. As shown in Figure 10b, both *RMSE_a_* and *RMSE_f_* decrease then increase with the radius of the circle increasing. The trends of the two curves are also roughly the same. When *r* is 6.8°, both *RMSE_a_* and *RMSE_f_* touch the bottom. The circle *r* equals 6.8° is drawn in dotted black line in Figure 10a. The third assumptive nominal angle is (50°, 42°), which is shown in Figure 11a. *RMSE_a_* and *RMSE_f_* changing with *r* are shown in Figure 11b. When *r* is 13.4°, both *RMSE_a_* and *RMSE_f_* touch the bottom. The circle *r* equals 13.4° is drawn in dotted black line in Figure 11a. The circles *r*, which equals 8.5°, 6.8° and 13.4° in the first, second and third trails, have common characteristics, such as initial positions of Gaussian components in these circles are within the given distributed sources with greater probability than any other circles. It is probable that estimation with initial positions of Gaussian components near the positions of Gaussian components in the given source has better accuracy than other parts, where the initial positions are far from the given source.

## 5. Conclusions

In this paper, we described the problem of estimating a 2D non-symmetric ID source based on L-shape arrays. The method we thus propose is developed by modeling the 2D non-symmetric APDF as a Gaussian mixture model. The estimation algorithm of a 2D non-symmetric ID source on the basis of iterative EM framework has been introduced in detail. The computational cost of a 2D non-symmetric ID source is much higher, when compared to the estimation of a 2D symmetric ID source. To evaluate the performance of estimation, we defined two indexes to reflect nominal angles and indicate spatial distribution. We investigated the parameter variety process of each Gaussian component, different *SNR*, number of snapshots, number of Gaussian components and initial positions of Gaussian components; the results of the numerical simulations show that the proposed method is effective for estimation of a non-symmetric ID source. 

## Figures and Tables

**Table 1:** Computational complexity of different methods.

Method	Calculation of the Sample Covariance Matrix	Searching/AP Algorithm	Eigendecomposition/Calculation of Weights	Total
Proposed	o (4 *NK*^2^) + o(4 *MK*^3^)	o(8 *MK*^3^)	o(8 *MK*^3^)	o (64 *MK*^3^) + o (4 *NK*^2^)
COMET	o (4 *NK*^2^)	o (24 *K*^3^ + 12 *K*^2^)		o (24 *K*^3^ + 12 *K*^2^) + o (4 *NK*^2^)
DISPARE	o (4 *NK*^2^)	o (24 *K*^2^+6 *K*)	o (8*K*^3^)	o (8 *K*^3^) + o (4 *NK*^2^) + o (24 *K*^2^ + 6 *K*)

## Appendix A

Considering that the random complex ray gain of the *l*th scatterer α*_l_*(t) has a property described by Equation (5), as scatterers have density distribution *f*(*θ*,*φ*), when *L* is a large number, Equation (13) can be expressed as:

(A1) $${\left[R_{x}\right]}_{kh}=E[x_{k}(t)x_{h}^{*}(t)]={\left|s(t)\right|}^{2}E\left[\sum_{l=1}^{L}\alpha_{l}(t)e^{j2\pi d(k-1)\cos\theta_{l}\sin\varphi_{l}/\lambda }\sum_{l^{\prime }=1}^{L}\alpha_{l^{\prime }}^{*}(t)e^{-j2\pi d(h-1)\cos\theta_{l^{\prime }}\sin\varphi_{l^{\prime }}/\lambda }\right]$$

*α_l_*(*t*) is supposed to be white with zero mean and independent from snapshot to snapshot as well as from scatterer to scatterer. $E[\alpha_{l}(t)\alpha_{l^{\prime }}^{*}(t^{\prime })]=\frac{\sigma_{\alpha }}{L}\delta (l-l^{\prime })\delta (t-t^{\prime })$ and $E[\alpha_{l}(t)\alpha_{l}(t^{\prime })]=0$, which is described by Equation (5). We can obtain:

(A2) $$E\left[\alpha_{l}(t)e^{j2\pi d(k-1)\cos\theta_{l}\sin\varphi_{l}/\lambda }\alpha_{l^{\prime }}^{*}(t)e^{-j2\pi d(h-1)\cos\theta_{l^{\prime }}\sin\varphi_{l^{\prime }}/\lambda }\right]=0,if\;l\neq l^{\prime }.$$

Then [**R***_x_*]*_kh_* can be expressed as:

(A3) $$\begin{array}{l} {\left[R_{x}\right]}_{kh}={\left|s(t)\right|}^{2}E\left[\sum_{l=1}^{L}\alpha_{l}(t)e^{j2\pi d(k-1)\cos\theta_{l}\sin\varphi_{l}/\lambda }\alpha_{l}^{*}(t)e^{-j2\pi d(h-1)\cos\theta_{l}\sin\varphi_{l}/\lambda }\right]\\ =\frac{\sigma_{\alpha }}{L}{\left|s(t)\right|}^{2}E\left[\sum_{l=1}^{L}e^{j2\pi d(k-h)\cos\theta_{l}\sin\varphi_{l}/\lambda }\right] \end{array}$$

As *f*(*θ*,*φ*) is the density function of scatterers of a source, we have:

(A4) $$E\left[e^{j2\pi d(k-h)\cos\theta_{l}\sin\varphi_{l}/\lambda }\right]=\iint e^{j2\pi d(k-h)\cos\theta \sin\varphi /\lambda }f(\theta ,\varphi )d\theta d\varphi .$$

Then [**R***_x_*]*_kh_* can be expressed as:

(A5) $$\begin{array}{l} {\left[R_{x}\right]}_{kh}=\frac{\sigma_{\alpha }}{L}{\left|s(t)\right|}^{2}E\left[\sum_{l=1}^{L}e^{j2\pi d(k-h)\cos\theta_{l}\sin\varphi_{l}/\lambda }\right]\\ =\sigma_{\alpha }{\left|s(t)\right|}^{2}\iint e^{j2\pi d(k-h)\cos\theta \sin\varphi /\lambda }f(\theta ,\varphi )d\theta d\varphi \end{array}$$

## Appendix B

Check **R***_x_* of Equation (16). According to Equation (14), the *k*th and *h*th elements of **a**(*θ,φ*) can be expressed as:

(A6) $${\left[a(\theta ,\varphi )\right]}_{k}=e^{j2\pi (k-1)d\cos\theta \sin\varphi /\lambda },$$

(A7) $${\left[a(\theta ,\varphi )\right]}_{h}=e^{j2\pi (h-1)d\cos\theta \sin\varphi /\lambda }.$$

We can obtain:

(A8) $${\left[a(\theta ,\varphi )a^{H}(\theta ,\varphi )\right]}_{kh}=e^{j2\pi (k-h)d\cos\theta \sin\varphi /\lambda }.$$

From Equation (13), the (*k*,*h*)th noise free element of covariance matrix **R***_x_* can be expressed as ${\left[R_{x}\right]}_{kh}=P\iint e^{j2\pi d(k-h)\cos\theta \sin\varphi /\lambda }f(\theta ,\varphi )d\theta d\varphi$. According to Equation (A9), the noise free [**R***_x_*]*_kh_* can be written as follows:

(A9) $${\left[R_{x}\right]}_{kh}=P\iint {\left[a(\theta ,\varphi )a^{H}(\theta ,\varphi )\right]}_{kh}f(\theta ,\varphi )d\theta d\varphi .$$

Considering the noise vector described by Equation (9), **R***_x_* can be expressed as follows:

(A10) $$R_{x}=P\iint f(\theta ,\varphi )a(\theta ,\varphi )a^{H}(\theta ,\varphi )d\theta d\varphi +\rho I_{K}.$$

The derivation processes of **R***_y_*, **R***_xy_* and **R***_yx_* is similar to **R***_x._*

## Appendix C

Check ${\left[r_{x}(u_{i})\right]}_{\mathrm{kh}}$ of Equation (27). Change variables $\left(\theta_{i}+\tilde{\theta }\right)$ for *θ* and $\left(\varphi_{i}+\tilde{\varphi }\right)$ for *φ*, where $\tilde{\theta }$ and $\tilde{\varphi }$ are the small deviation of $\theta_{i}$ and $\varphi_{i}$. Thus, sin*θ*sin*φ* and cos*θ*sin*φ* can be approximated by the first term in the Taylor series expansions:

(A11) $$\begin{array}{l} {\left[r_{x}(u_{i})\right]}_{kh}\approx \iint \frac{1}{2\pi \sigma_{\theta i}\sigma_{\varphi i}}e^{-0.5\left[{\left(\frac{\tilde{\theta }}{\sigma_{\theta i}}\right)}^{2}+{\left(\frac{\tilde{\varphi }}{\sigma_{\varphi i}}\right)}^{2}\right]}e^{j\pi (k-h)\left(\cos\theta_{i}\sin\varphi_{i}+\cos\theta_{i}\cos\varphi_{i}\tilde{\varphi }-\sin\theta_{i}\sin\varphi_{i}\tilde{\theta }\right)}d\tilde{\theta }d\tilde{\varphi }\\ =e^{j\pi (k-h)\cos\theta_{i}\sin\varphi_{i}}\iint \frac{1}{2\pi \sigma_{\theta i}\sigma_{\varphi i}}e^{-0.5\left[{\left(\frac{\tilde{\theta }}{\sigma_{\theta i}}\right)}^{2}+{\left(\frac{\tilde{\varphi }}{\sigma_{\varphi i}}\right)}^{2}-2j\pi (k-h)(\cos\theta_{i}\cos\varphi_{i}\tilde{\varphi }-\sin\theta_{i}\sin\varphi_{i}\tilde{\theta })\right]}d\tilde{\theta }d\tilde{\varphi }\\ =e^{j\pi (k-h)\cos\theta_{i}\sin\varphi_{i}}e^{-0.5\left\{{\left[\pi \sigma_{\theta i}(k-h)\sin\theta_{k}\sin\varphi_{i}\right]}^{2}+{\left[\pi \sigma_{\varphi i}(k-h)\cos\theta_{i}\cos\varphi_{i}\right]}^{2}\right\}} \end{array}.$$

## Appendix D

Assume that complete data **z***_i_*(*t*) and incomplete data **z**(*t*) are zero-mean complex Gaussian random vectors and their covariance matrices are **R**_zi_ and **R**_z_, respectively. According to the incoherently distributed source assumption, different vectors **z***_i_*(*t*) are uncorrelated. Since **z**(*t*) is a linear combination of uncorrelated **z***_i_*(*t*), the covariance matrix of **z***_i_*(*t*) and **z**(*t*) can be written as follows:

(A12) $$E[z_{i}(t)z^{*}(t)]=E[z_{i}(t)z_{i}^{*}(t)]=R_{zi}.$$

Thus, the conditional expectation and variance of **z***_i_*(*t*) under given observed data can be expressed as follows:

(A13) $$E[z_{i}(t)|z(t)]=0+E[z_{i}(t)z^{*}(t)]R_{{}_{z}}^{-1}[z(t)-0]=R_{zi}R_{{}_{z}}^{-1}z(t).$$

(A14) $$D[z_{i}(t)|z(t)]=R_{zi}-E[z_{i}(t)z^{*}(t)]R_{{}_{z}}^{-1}E[z_{i}(t)z^{*}(t)]=R_{zi}-R_{zi}R_{{}_{z}}^{-1}R_{zi}.$$

Thus, the conditional expectation of covariance matrix **R**_zi_ under the given observed data can be expressed as follows:

(A15) $$E[{\hat{R}}_{zi}|{\hat{R}}_{z}]=R_{zi}{\left(R_{z}\right)}^{-1}{\hat{R}}_{z}{\left(R_{z}\right)}^{-1}R_{zi}+R_{zi}-R_{zi}{\left(R_{z}\right)}^{-1}R_{zi}$$
